# Effects of strengthening, stretching and functional training on foot function in patients with diabetic neuropathy: results of a randomized controlled trial

**DOI:** 10.1186/1471-2474-15-137

**Published:** 2014-04-27

**Authors:** Cristina D Sartor, Renata H Hasue, Lícia P Cacciari, Marco K Butugan, Ricky Watari, Anice C Pássaro, Claudia Giacomozzi, Isabel CN Sacco

**Affiliations:** 1 Physical Therapy, Speech and Occupational Therapy Dept, School of Medicine, University of São Paulo, 51, Cidade Universitária, São Paulo, SP, Brazil; 2 Department of Technology and Health, Italian National Institute of Health, Rome, Italy

**Keywords:** Diabetic neuropathies, Rehabilitation, Exercise therapy, Walking, Randomized controlled trial, Foot, Ankle, Musculoskeletal manipulation

## Abstract

**Background:**

Foot musculoskeletal deficits are seldom addressed by preventive medicine despite their high prevalence in patients with diabetic polyneuropathy.

AIM: To investigate the effects of strengthening, stretching, and functional training on foot rollover process during gait.

**Methods:**

A two-arm parallel-group randomized controlled trial with a blinded assessor was designed. Fifty-five patients diagnosed with diabetic polyneuropathy, 45 to 65 years-old were recruited. Exercises for foot-ankle and gait training were administered twice a week, for 12 weeks, to 26 patients assigned to the intervention group, while 29 patients assigned to control group received recommended standard medical care: pharmacological treatment for diabetes and foot care instructions. Both groups were assessed after 12 weeks, and the intervention group at follow-up (24 weeks). Primary outcomes involved foot rollover changes during gait, including peak pressure (PP). Secondary outcomes involved time-to-peak pressure (TPP) and pressure–time integral (PTI) in six foot-areas, mean center of pressure (COP) velocity, ankle kinematics and kinetics in the sagittal plane, intrinsic and extrinsic muscle function, and functional tests of foot and ankle.

**Results:**

Even though the intervention group primary outcome (PP) showed a not statistically significant change under the six foot areas, intention-to-treat comparisons yielded softening of heel strike (delayed heel TPP, p=.03), better eccentric control of forefoot contact (decrease in ankle extensor moment, p<.01; increase in function of ankle dorsiflexion, p<.05), earlier lateral forefoot contact with respect to medial forefoot (TPP anticipation, p<.01), and increased participation of hallux (increased PP and PTI, p=.03) and toes (increase in PTI, medium effect size). A slower COP mean velocity (p=.05), and an increase in overall foot and ankle function (p<.05) were also observed. In most cases, the values returned to baseline after the follow-up (p<.05).

**Conclusions:**

Intervention discreetly changed foot rollover towards a more physiological process, supported by improved plantar pressure distribution and better functional condition of the foot ankle complex. Continuous monitoring of the foot status and patient education are necessary, and can contribute to preserving the integrity of foot muscles and joints impaired by polyneuropathy.

**Trial registration:**

ClinicalTrials.gov Identifier: NCT01207284, registered in 20^th^ September 2010.

## Background

The increased risk of plantar ulceration in patients with diabetic polyneuropathy (DPN) is often associated with a heterogeneous plantar pressure distribution characterized by overloading of anterior regions, unloading of toes and hallux [1-6], and a reduced role of lateral forefoot and toes in the foot rollover during stance phase [7]. This loading pattern is a result of alterations in the foot rollover process during walking [7-9], following an overall worsening of foot-ankle muscular, articular and nervous function.

From a biomechanical point of view, the main normal foot functions during walking are to dampen down heel stress by reducing excessive impulse loading upon initial contact, adapt to the instantaneous changes in contact surface during midstance, and provide stability during propulsion [10,11]. As DPN progresses, the integrity is affected of the muscles and neural structures [12,13] mostly of the small joints of the foot and ankle [8], which restricts the proper load absorption and management in the gait stance phase. Sustained hyperglycemia causes additional structural alterations which affect the physiological gait biomechanics —impaired tropism and activation of muscles—which interfere with the quality and control of movement, drastically affecting load absorption and transmission during the foot rollover process [14,15]. More specifically, Tibialis anterior weakness could potentially contribute to higher peak pressures [16-18], and the resulting atrophy of the small intrinsic foot muscles [19,20] could compromise the static and dynamic stability of foot rollover.

Unfortunately, there is still no efficient and definitive intervention to manage this epidemic, chronic complication (DPN), which highlights the relevance of studying complementary and synergistic treatments and/or reinforcing preventive actions. DPN is an insidious, long-term complication, thus preventive actions are essential in the care of the population with diabetes.

Since higher peak pressure values have been correlated with an increased incidence of plantar ulceration [1,2], the reduction of tissue stress has been established as one of the main goals of interventions in DPN patients. Offloading techniques have been extensively explored in the literature, in particular under the form of custom-made footwear, insoles, and orthotic devices. The effectiveness of this approach, however, is hard to confirm: standardization is lacking and the application of the appropriate techniques is not always assured [21,22], not to speak of the very low adherence to this type of treatment [23].

Offloading devices, whose main target is to redistribute plantar loads by acting as an external device at the interface between the foot and the ground, may passively induce, as a minor consequence, some changes in the musculoskeletal control of the foot rollover. Conversely, foot and ankle exercises have the main aim, and the benefit, of changing the foot rollover actively as a major consequence of their being performed on a regular basis, thereby promoting a proper musculoskeletal response, i.e. absorbing and transmitting loads as the body moves forward during walking.

Few authors have described the subtle, positive effects of foot and ankle exercises in this population, although some studies were not randomized controlled trials [24,25], and others reported on quite short interventions and follow-up periods [26,27]. Therefore, there are still not enough data for conclusions to be arrived at regarding the effectiveness of such interventions.

The present study describes the outcomes of a randomized, controlled trial to describe the effects of active stretching, strengthening, balance, and walking exercises [28] on the foot rollover process of DPN patients with no previous episode of plantar ulceration.

The main hypothesis of the study was that a physiotherapeutic intervention would improve the foot rollover process and successfully redistribute dynamic plantar loading. The complex dynamic process of foot rollover is hardly representable by means of a single variable, but it can be described by a combination of plantar pressure variables such as peak pressure, pressure–time integral, center of pressure trajectory and velocity. We chose peak pressure as the primary outcome because it is the most commonly described one for the population investigated [1,29-31]. However, the description of foot rollover process was also accomplished using other related biomechanical (kinetic and kinematic parameters) and clinical variables hereby referred as secondary outcomes.

The clinical relevance of this study relies on the assumption that this specific intervention could lead to the recovery of muscle and joint function in patients with DPN, and enable them to maintain, for as long as possible, the residual capability to interact safely with the ground while walking or standing.

## Methods

The clinical trial was approved by the local Ethics Committee (Comitê de Ética em Pesquisa da Faculdade de Medicina da Universidade de São Paulo, protocol number 054/10). All patients gave their written informed consent according to the standard forms. The study started in August 2010 but the recruitment period lasted from October 2010 to August 2012.

### Setting and participants

The eligibility criteria were: patients 45–65 years of age; diabetes mellitus type 1 or 2; diagnosed for at least seven years; body mass index ranging 18.5–29.9 kg/m^2^ (normal and overweight classifications); DPN diagnosed by the medical care center; score higher than 2 out of a maximum of 13 points in the Michigan Neuropathy Screening Instrument (MNSI) questionnaire [32], indicating the presence of at least two DPN symptoms; score greater than 1 point on a 10 point scale for physical assessment according to the MNSI instrument, but always including impaired vibration perception; ability to walk independently in the laboratory; without any episode of plantar ulceration no partial or total foot amputation; and not receiving any physiotherapy intervention or offloading devices. Patients were not included if they had other neurological or orthopedic impairments, major vascular complications, severe retinopathy, or severe nephropathy.

The baseline characteristics of the participants are provided in Table 1.

**Table 1:** Baseline demographic and clinical characteristics of each group

	**Control Group (n = 29)**	**Intervention Group (n = 26)**	**p (T-test)**	**p (Mann–Whitney)**
Age (years)	60 (12)	59 (4)	0.40	-
Sex (% female)	52	42	-	0.34
Time of onset of diabetes (years)	18 (11)	17 (10)	0.81	-
Type 2 diabetes (%)	92.9	96.9	-	0.85
Fasting blood glucose (mg/dL)	166.7 (98.6)	157.6 (89.8)	0.60	-
Body mass (kg)	82.5 (16.4)	77.4 (14.1)	0.24	-
Height (m)	1.71 (0.30)	1.65 (0.09)	0.30	-
BMI (kg/m^2^)	29 (4)	28 (4)	0.54	-
Insulin intake (%)	60	50	-	0.61
Practice regular physical activity	33%	42%		-
MNSI^a^ questionnaire (median)	6 [3;8]	6 [4;8]	-	0.59
MNSI^a^ physical assessment (median)	4.5 [3.0;6.5]	4.5 [3.0;6.5]	-	0.62

^a^Michigan Neuropathy Screening Instrument.

The participants were recruited from three settings: (a) a diabetes mellitus ambulatory medical care located in a regional hospital, (b) the National Association of Diabetes (ANAD), and (c) a primary care center at the School of Medicine of the University. Candidates for recruitment were interviewed on the telephone. If selected, they went through a first assessment to confirm the eligibility criteria. This first assessment was taken as the baseline condition. The physical therapist who performed all the clinical assessments was blinded to the groups.

The patients allocated to the intervention group were treated in the Physical Therapy Department of the University, in a real ambulatory setting.

### Design overview

**Figure 1:**
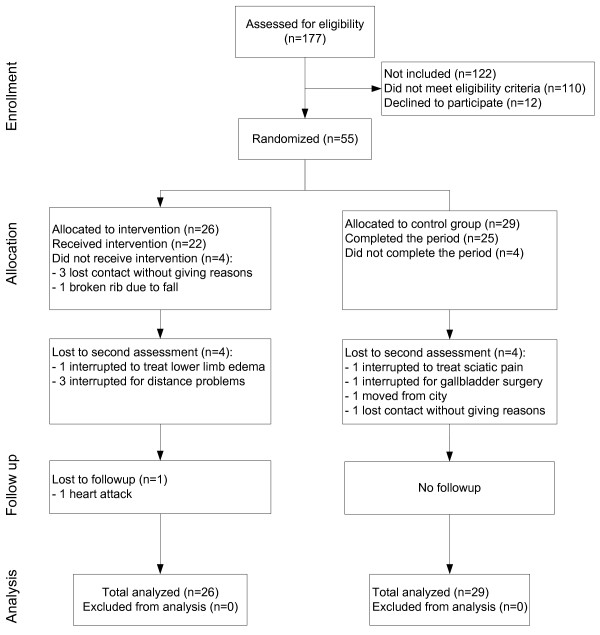
Flow of participants through the trial.

A two-arm, parallel-group randomized controlled trial was designed. The patients allocated to the intervention group (IG) received physical therapy and instructions to perform exercises at home; the control group (CG) received neither. Both groups were assessed at baseline and after 12 weeks. The IG was further assessed at week 24 (follow-up period). Initially, we planned to implement a crossover arm for the CG in order to achieve the sample size as early as possible [28]. Unfortunately, there was a high incidence of non-adherence of these patients to receive intervention after the 12 weeks, and the crossover intervention could not be implemented. Only 8 out of the 29 patients initially allocated to CG, confirmed their interest in receiving intervention after 12 weeks. Because of this low adherence and the ensuing cancellation of the crossover arm, it was not possible to assess the CG together with the IG in the follow-up evaluation. The final design of the study is presented in Figure 1.

### Randomization and interventions

The randomization schedule was done with Clinstat software [33] by an independent researcher, who was not aware of group codes. A numeric block randomization sequence, with blocks varying randomly in size from one to eight, was kept in sequentially numbered, opaque envelopes. After an initial assessment for compliance with eligibility criteria, patient enrollment was conducted by a physical therapist who also performed all the blind assessments. If a patient agreed to participate in the study, allocation to a group was made by a second independent researcher, unaware of group codes. Only the physiotherapist who administered the intervention did know who should receive it.

The intervention - 40–60 minutes per session, twice a week, for 12 weeks - started as soon as the patient was allocated to the IG. The therapeutic session was divided into four blocks of exercises with the following aims: (a) increase foot and ankle range of motion, (b) strengthen foot and ankle muscles, (c) increase foot and ankle performance through functional exercises, and (d) increase walking skills and foot rollover training. Each session included a selection of exercises from each of the four exercises groups. The patient was exposed to gradual and progressive difficulty; attention was paid to pain and/or performance deterioration during execution. In each session, the exercises followed a sequence that started with passive exercises, progressed to active ones, and finished with walking and functional skills. Therefore, the motor integration of peripheral gains into functional movements could be optimized during each session. The complete intervention protocol is described in Additional file 1: Table S1 and has been published elsewhere [28]. Some images of the exercises are shown in Additional file 2: Figure S2 that were available by a written informed consent provided by the patients.

The CG did not receive any Physical Therapy intervention or any instruction to perform exercises at home during the same period, but continued to receive customized medical care, which included pharmacological treatment for diabetes and foot care instructions as recommended by Bakker et al. [34]. The dropouts and declines are described in the flow chart of the participants (Figure 1).

Apart from intervention exercises, neither group changed the amount of physical activity after the beginning of the study.

### Primary outcomes

Although the foot rollover process during gait may be represented by a combination of plantar loading variables, we chose peak pressure (PP) over plantar surface as primary outcome. For the purpose of sample size calculation, the peak pressure under the lateral forefoot was chosen because it is typically one of the main foot areas that present alterations due to DPN [7] and has been demonstrated to be different substantially from healthy individuals [31].

### Secondary outcomes

The secondary outcomes were grouped in three sets: (1) additional loading variables representing foot rollover; (2) kinematic and kinetic variables of the ankle joint; (3) clinical outcomes.

The loading variables were: (a) mean velocity of the total trajectory of the center of pressure (COP), which indicates how fast the body moves over the foot, (b) time to peak pressure (TPP), which detects whether the contact of a specific foot area is anticipated or delayed after the intervention, (c) pressure–time integral (PTI) over six plantar areas (heel, midfoot, lateral and medial forefoot, hallux and toes).

The kinematic and kinetic outcomes of the ankle joint were: (a) the total sagittal range of motion during the stance phase of gait (degrees), (b) the joint angle (sagittal plane) at the end of the propulsion phase (degrees), (c) the peak of extensor and flexor moments at approximately 20% and 80% of the stance phase of gait (% body weight* height), corresponding to the flattening of the foot and the beginning of propulsion, respectively.

The clinical outcomes were: (a) foot and ankle muscle function, (b) functional tests for foot and ankle, (c) scores of Michigan Neuropathy Screening Instrument (MNSI questionnaire and foot physical assessment) [32], (d) the score for the Activities-specific Balance Confidence (ABC) Scale [35].

### Data acquisition of all outcomes and mathematical analysis of the biomechanical outcomes

#### Loading variables

Plantar pressure distribution was recorded using the Pedar-X System (Novel, Munich, Germany) at 100 Hz. The patients walked barefoot on a 10 m walkway at a self-selected cadence resulted within 96–116 steps/min (normal gait cadence) [36], with the insole placed and fixed, using anti-skid socks fixed with a stripe around the ankle. Twenty five valid steps per foot (the middle steps of 4 trials) were acquired and analyzed for each patient. The plantar pressure data (PP, PTI, and COP mean velocity) were calculated for 6 areas of interest (heel, midfoot, medial forefoot, lateral forefoot, hallux and toes) using the software Novel-projects (Novel, Munich, Germany). The plantar surface was first divided into three larger areas: rearfoot (27% of foot length), midfoot (28% of foot length), and forefoot and toes (45% of foot length). The forefoot was subdivided in width into: medial forefoot (55% of the forefoot width) and lateral forefoot (45% of the forefoot width). The forefoot was also subdivided in length into: hallux (the final 20% of foot length with 33% of width) and toes (the final 20% of foot length with 67% of width). TPP was calculated in a custom-written MATLAB function from the peak pressure temporal series as the instant of peak occurrence relative to stance duration (also for the same 6 foot areas).

#### Kinematic and kinetic outcomes

The kinematics of retro-reflective markers, with attachment locations based on the Cleveland protocol [37], were recorded at 100 Hz by six infrared cameras (2D accuracy < 2 mm) (OptiTrack FLEX: V100, Natural Point, Corvallis, OR, USA) [38] using AMASS^TM^ software (C-Motion, USA). Ground reaction force data were acquired at 100 Hz by means of a strain gauge force plate (AMTI OR-6 – 1000, Watertown, MA, USA). For the ground reaction force and kinematic data acquisition, patients walked barefoot, at the same walking cadence established at plantar pressure trials, and ten valid trials were acquired and analysed for each patient. The kinematic and ground reaction force data were synchronized and sampled at 100 Hz by an A/D card (AMTI, DT 3002, 12 bits). Data were filtered with low pass filter Butterworth of 4^th^ order at 20 Hz for force data and 6 Hz for kinematics. The bottom-up inverse dynamic method was used for calculating the net ankle moment. The human body was modeled by three linked segments (foot, shank and thigh), and the anthropometric parameters were adopted from Dempster & Gaughran [39]. Mathematical analyses of the kinematic and ground reaction force data were performed using Visual3D software (C-motion, Kingston, ON, Canada), and the net ankle moment of force was calculated in a custom-written Matlab function (MathWorks, Natick, MA, USA).

#### Clinical outcomes

All the clinical assessments were performed by a physiotherapist who was blind to group allocation. They included anamnesis for personal details and diabetes history, MNSI scores and ABC score.

Intrinsic and extrinsic foot and ankle muscle functions were assessed with manual function testing [40,41], as there is currently no available instrument capable of measuring the functions of tested muscles. The assessed muscles were triceps surae, tibialis anterior, interosseous, lumbrical, flexor hallucis brevis, flexor digitorum brevis, extensor hallucis longus and brevis, and extensor digitorum longus and brevis.

For the evaluation of the feet and ankle function, we used functional tests based on Magee et al. (1997), in which the patients were asked to perform the following movements: ankle flexion and extension in one leg stance and toes flexion and extension in sitting position. Patients were asked to repeat each movement as fast as possible for 30 seconds, without compensating the movements. Classification was according to the following scale: 0 repetitions: not functional; 1–4 repetitions: barely functional; 5–9 repetitions: reasonably functional; 10–15 repetitions: functional.

### Statistical analysis

Sample size was calculated on the basis of the primary outcome (PP at the lateral forefoot) and was accomplished using a moderate effect size (f = 0.36). Standard deviation estimates were taken from one of the authors’ previous studies, wherein a similar patient cohort had been recruited [42]. A sample size of 46 subjects initially was calculated to detect a moderate effect difference between the highest and lowest group pressure means, with a power of 81%, an alpha level of 0.05, a statistical design of F test of repeated measures (between and within effects), and assuming a 10% loss to follow-up. The actual loss of the study was of 15.6% of patients; sample size was, therefore, recalculated and a total of 55 patients were recruited to warrant sample power.

An intention-to-treat analysis was performed. The missing data was treated as missing completely at random (MCAR) [43], using the ‘mean of series’ imputation method [44] for the values with normal distribution (TPP, PP, PTI, mean COP velocity, kinetic and kinematic variables, MNSI questionnaire score and ABC score) and using the median for non-parametric variables (muscle function tests, functional tests and MNSI scores for physical assessment).

After the confirmation of normality (Kolmogorov–Smirnov test) and homoscedasticity (Levene’s test) of parametric variables, a casewise two-way ANOVA for repeated measures, followed by a post hoc Newman–Keuls correction, were used to verify the interaction effect of Group x Time between baseline and after 12 weeks (p < 0.05). Comparisons between baseline and 12-week and 24-week follow-up were applied only to the IG, using ANOVA for repeated measures or Friedman’s test to verify whether patients who had received the intervention returned to the baseline condition (p < 0.05). Non-parametric data were compared using Mann–Whitney tests for between-groups comparisons and Wilcoxon tests for time effect comparisons (p < 0.05).

To describe the intervention effect, Cohen’s d coefficients were calculated, as well as mean or median differences, and its 95% confidence interval.

### Role of the funding source

This study was funded by Sao Paulo Research Foundation (FAPESP, process number 2011/19304-4) and by Conselho Nacional de Desenvolvimento Científico e Tecnológico (CNPq, process number 556374/2010-0). The funding sources had no role in study design, data collection, data analysis, interpretation of data, or writing of the trial report. The investigators had final responsibility in the decision to submit the report for publication. The study was prospectively registered at ClinicalTrials.gov (NCT01207284).

## Results

The flowchart of the participants who completed or abandoned the intervention and follow-up periods is described in Figure 1. A total of 26 patients went into the IG, and 29 into the CG. The participants did not differ at baseline with respect to demographic, diabetes, and foot characteristics (Table 1). For most of the loading variables, mean velocity of COP displacement, and muscle function, the groups did not differ at baseline, except for PP at heel and medial forefoot (Table 2), muscle function of extensor digitorum longus and brevis, and toes extension on functional tests (Table 3).

**Table 2:** Mean, standard deviations and effects of intervention and follow-up on pressure variables

**Time-to-peak pressure (% of stance phase)**									
**Foot areas**	**Control group**		**Intervention group**			**Intervention effect**		**Follow-up effect**	
	**Baseline (*a *) n = 29**	**12 weeks (*b *) n = 29**	**Baseline (*c *) n = 26**	**12 weeks (*d *) n = 26**	**24 weeks (*e *) n = 26**	**Effect size**^***b-d ***^**(Cohen’s d)**	**p**^***a-b-c-d ***^**(interaction)**	**Effect size (Cohen’s d)**	**p**^***c-d-e***^
**Heel**	17.9 (6.0)	18.1 (5.5)	17.4^*‡^ (5.4)	19.9^*‡^ (3.7)	17.7^‡^ (4.6)	0.3 (small)	0.03	0.5 (medium)^c-d^	<0.01
								0.1 (small)^c-e^	
**Midfoot**	54.2 (14.3)	53.5 (13.6)	51.2 (16.6)	50.5 (15.2)	46.6 (12.7)	0.2 (small)	0.98	0.0 (small)^c-d^	0.12
								0.3 (small)^c-e^	
**Medial forefoot**	81.9 (4.4)	82.1 (2.3)	82.0 (4.6)	81.6 (4.1)	81.7 (3.3)	0.1 (small)	0.52	0.1 (small)^c-d^	0.69
								0.1 (small)^c-e^	
**Lateral forefoot**	80.3 (4.6)	80.7 (3.1)	82.0^*¶^ (3.7)	80.4^*^ (3.5)	80.3 (3.2)	0.1 (small)	0.01	0.5 (medium)^c-d^	<0.01
								0.5 (medium)^c-e^	
**Hallux**	85.8 (5.2)	85.6 (3.6)	85.0 (9.9)	83.4 (10.2)	84.8 (7.0)	0.3 (small)	0.18	0.2 (small)^c-d^	0.17
								0.0 (small)^c-e^	
**Toes**	83.5 (6.0)	83.1 (4.0)	83.2 (9.9)	81.5 (9.8)	82.5 (3.8)	0.2 (small)	0.36	0.2 (small)^c-d^	0.39
								0.1 (small)^c-e^	
**Center of pressure – mean velocity (m/s)**									
**Heel**	0.4 (0.13)	0.4 (0.11)	0.4 (0.1)	0.4 (0.1)	0.4 (0.1)	0.1 (small)	0.30	0.1 (small)^c-d^	0.44
								0.0 (small)^c-e^	
**Midfoot**	0.4 (0.11)	0.5^†^ (0.11)	0.5^¶^ (0.1)	0.4^†^ (0.1)	0.4 (0.1)	0.4 (medium)	0.03	0.2 (small)^c-d^	<0.01
								0.5 (medium)^c-e^	
**Medial forefoot**	0.3 (0.09)	0.3 (0.07)	0.3^#^ (0.1)	0.3 (0.1)	0.3^#^ (0.1)	0.0 (small)	0.90	0.3 (small)^c-d^	<0.01
								0.6 (medium)^c-e^	
**Lateral forefoot**	0.3^§^ (0.17)	0.3 (0.11)	0.3^¶^ (0.1)	0.2^§^ (0.1)	0.2 (0.1)	0.3 (small)	0.58	0.4 (medium)^c-d^	<0.01
								0.6 (medium)^c-e^	
**Hallux**	0.2 (0.23)	0.2^||^ (0.27)	0.1^||^ (0.2)	0.1^||^ (0.1)	0.2^¶^ (0.2)	0.5 (medium)	0.27	0.1 (small)^c-d^	<0.01
								0.4 (medium)^c-e^	
**Toes**	0.1 (0.18)	0.1 (0.14)	0.1 (0.1)	0.1 (0.2)	0.1 (0.1)	0.2 (small)	0.95	0.1 (small)^c-d^	0.15
								0.3 (small)^c-e^	
**Total**	0.3 (0.05)	0.4 (0.04)	0.4 (0.1)	0.3 (0.0)	0.3 (0.0)	0.2 (small)	0.05	0.3 (small)^c-d^	<0.001
								0.7 (large)^c-e^	
**Pressure time integral (kPa.s)**									
**Heel**	79.1 (22.3)	79.1 (17.3)	81.0 (26.8)	87.6^¶^ (23.8)	77.9 (17.4)	0.4 (medium)	0.11	0.3 (small)^c-d^	<0.01
								0.1 (small)^c-e^	
**Midfoot**	42.9^*^ (20.9)	38.8^*^ (20.6)	39.2 (14.7)	42.8 (25.4)	31.7^¶^ (9.1)	0.2 (small)	0.03	0.2 (small)^c-d^	<0.01
								0.6 (medium)^c-e^	
**Medial forefoot**	90.2 (28.3)	93.8 (20.5)	101.2 (29.9)	110.6^†¶^ (27.4)	103.7 (25.7)	0.7 (medium)	0.20	0.3 (small)^c-d^	0.01
								0.1 (small)^c-e^	
**Lateral forefoot**	90.9 (24.6)	92.6 (20.5)	92.4 (22.4)	98.7^¶^ (24.6)	88.2 (19.7)	0.3 (small)	0.29	0.2 (small)^c-d^	<0.01
								0.2 (small)^c-e^	
**Hallux**	48.6 (22.6)	50.6 (19.2)	47.2^‡#^ (21.4)	55.1^‡#^ (19.3)	49.5 (24.6)	0.2 (small)	0.22	0.4 (medium)^c-d^	0.03
								0.1 (small)^c-e^	
**Toes**	48.0 (24.1)	44.5 (22.6)	50.6 (22.9)	55.6 (26.9)	52.6 (21.4)	0.5 (medium)	0.16	0.2 (small)^c-d^	0.44
								0.1 (small)^c-e^	
**Peak pressure (kPa)**									
**Heel**	293.6 (68.4)	305.4 (64.3)	314.1 (88.0)	324.1 (77.6)	293.4 (64.4)	0.3 (small)	0.91	0.1 (small)^c-d^	0.01
								0.3 (small)^c-e^	
**Midfoot**	125.1 (63.6)	113.1 (61.3)	119.5 (43.7)	122.3 (46.5)	91.7^¶^ (21.4)	0.2 (small)	0.08	0.1 (small)^c-d^	<0.001
								0.8 (large)^c-e^	
**Medial forefoot**	313.4^§^ (101.4)	328.7 (77.2)	356.3^§^ (100.1)	370.8^§^ (89.4)	350.5 (81.9)	0.5 (medium)	0.95	0.2 (small)^c-d^	0.16
								0.1 (small)^c-e^	
**Lateral forefoot**	297.9 (83.9)	307.1 (72.5)	316.8 (79.5)	318.5 (56.4)	291.1^¶^ (65.8)	0.2 (small)	0.59	0.0 (small)^c-d^	<0.01
								0.4 (large)^c-e^	
**Hallux**	214.8 (69.2)	229.7 (85.1)	206.9^‡^ (96.8)	235.2^‡¶^ (84.4)	212.1 (95.4)	0.1 (small)	0.46	0.1 (small)^c-d^	0.03
								0.0 (small)^c-e^	
**Toes**	180.6 (99.6)	173.9 (93.2)	187.1 (84.2)	199.0 (94.8)	185.3 (73.0)	0.3 (small)	0.40	0.16 (small)^c-d^	0.53
								0.01 (small)^c-e^	

^*^different conditions between each other (interaction and time effect p = 0.01).

^†^condition *b* is different from *d* (interaction effect).

^‡^conditions *c d,* and *e* are different from one another in follow-up comparisons.

^¶^the different condition from the others in follow-up comparisons (p < 0.01).

^§^condition *a* is different from *d* (time effect, p = 0.04).

^||^condition *b* is different from *c* and *d* (group effect, p < 0.01).

^#^condition *c* is different from *e* in follow-up comparisons (p < 0.01).

Letters a, b, c, d and e represent the groups conditions.

**Table 3:** Intervention and follow-up effects of clinical assessment

**Diabetic polineuropathy clinical assessment**							
**Instrument**	**Control Group**		**Intervention Group**			**Intervention effect**	**Follow-up effect**
	**Baseline (*a *) n = 29**	**12 weeks (*b *) n = 29**	**Baseline (*c *) n = 26**	**12 weeks (*d *) n = 26**	**24 weeks (*e *) n = 26**	**Effect size or median difference (CI 95%)**^**b-d**^	**Effect size or median difference (CI 95%)**^**b-d**^
**MNSI questionnaire (score)**^**a**^	6 (3)	6 (3)	6^¶§^ (2)	4^¶^ (3)	4 (2)	0.52 (medium)	0.54 (medium)^c-d^
							0.54 (medium)^c-e^
**MNSI physical assessment (score)**^**b**^	4.5 (3; 6.5)	5.5 (2.5; 7)	4.5 (3.0; 6.5)	4.0^§^ (4.0; 5.0)	5.0^§^ (5.0; 5.5)	-1.5 [-2.8 a -0.2]	-0.5 [-1.7 a 1.7]^c-d^
							0.5 [-0.7 a 1.7]^c-e^
**ABC questionnaire (% of confidence)**^**c**^	78 (18)	78 (19)	84 (16)	86^§^ (8)	89^§^ (8)	0.50 (medium)	0.34 (small)^c-d^
							0.21 (small)^c-e^
**Muscle function**^**d**^							
**Extensor digitorum longus and brevis**	3.5^*^	4.0	4.0^*§^	4.0	4.0^§^	0.0 [0.0 to 0.0]	0.0 [0.0 to 0.0]^c-d^
	(3.0; 4.0)	(3.0;5.0)	(3.3;5.0)	(4.0;4.0)	(3.0;4.0)		0.0 [0.0 to 0.0]^c-e^
**Extensor hallucis longus and brevis**	4.0	4.0	4.0	4.0	4.0	0.0 [0.0 to 0.0]	0.0 [-0.5 to 0.5]^c-d^
	(4.0;5.0)	(4.0;5.0)	(4.0;5.0)	(4.0;5.0)	(4.0;4.0)		0.0 [-0.5 to 0.5]^c-e^
**Flexor digitorum brevis**	4.0^†^	4.0^†‡^	4.0	5.0^‡^	4.0	-1.0 [-1.0 to -1.0]	0.0 [-0.5 to 0.5]^c-d^
	(4.0;5.0)	(3.0;5.0)	(3.0;5.0)	(4.0;5.0)	(4.0;4.0)		0.0 [-0.5 to 0.5]^c-e^
**Flexor hallucis brevis**	4.0	4.0	4.0	4.0	4.0	0.0 [0.0 to 0.0]	0.0 [0.0 to 0.0]^c-d^
	(3.0;5.0)	(3.3;4.8)	(4.0;5.0)	(4.0;5.0)	(4.0;4.0)		0.0 [0.0 to 0.0]^c-e^
**Lumbrical**	4.0^†^	3.0^†^	4.0	4.0^§^	3.5^§^	-1.0 [-1.5 to -.05]	0.0 [-0.9 to 0.9]^c-d^
	(3.0;5.0)	(2.0;5.0)	(2.0;5.0)	(3.0;4.8)	(2.3;4.0)		0.5 [-0.4 to 0.4]^c-e^
**Interosseous**	3.5	3.0^‡^	4.0	4.0^‡§^	3.0^§^	-1.0 [-1.5 to -0.5]	0.0 [0.5 to 1.5]^c-d^
	(2.0;4.0)	(2.0;4.0)	(2.0;5.0)	(3.0;4.0)	(3.0;3.0)		1.0 [0.5 to 1.5]^c-e^
**Tibialis anterior**	4.5	4.0^‡^	4.0	5.0^‡^	4.0	-1.0 [-1.0 to -1.0]	0.0 [-0.5 to 0.5]^c-d^
	(3.0;5.0)	(4.0;5.0)	(4.0;5.0)	(4.0;5.0)	(4.0;4.0)		0.0 [-0.5 to 0.5]^c-e^
**Triceps surae**	5.0	5.0	5.0^¶^	5.0^¶^	5.0	0.0 [0.0 to 0.0]	0.0 [0.0 to 0.0]^c-d^
	(4.0;5.0)	(4.0;5.0)	(4.0;5.0)	(5.0;5.0)	(4.0;5.0)		0.0 [0.0 to 0.0]^c-e^
**Functional tests**^**e**^							
**Ankle flexion**	3.5	3.0^‡^	4.0^¶^	4.0^‡¶#^	3.0	1.0 [0.5 a 1.5]	0.0 [-0.4 a 2.4]^c-d^
	(1.0;4.0)	(1.0;4.0)	(1.0;4.0)	(3.0;4.0)	(1.0;4.0)		1.0 [-0.4 a 2.4]^c-e^
**Ankle extension**	4.0^†^	4.0^†^	4.0^¶§^	4.0^¶^	4.0^§^	0.0 [0.0 a 0.0]	0.0 [0.0 a 0.0]^c-d^
	(4.0;4.0)	(4.0;4.0)	(4.0;4.0)	(4.0;4.0)	(4.0;4.0)		0.0 [0.0 a 0.0]^c-e^
**Toes flexion**	4.0	3.5^‡^	4.0^¶#^	4.0^‡¶^	4.0	0.5 [0.0 a 1.0]	0.0 [-0.5 a 0.5]^c-d^
	(3.0;4.0)	(3.0;4.0)	(3.0;4.0)	(4.0;4.0)	(4.0;4.0)		0.0 [-0.5 a 0.5]^c-e^
**Toes extension**	4.0^*^	4.0^‡^	4.0^*¶^	4.0^‡¶§^	4.0^§^	0.0 [0.0 a 0.0]	0.0 [0.0 a 0.0]^c-d^
	(3.3;4.0)	(4.0;4.0)	(4.0;4.0)	(4.0;4.0)	(4.0;4.0)		0.0 [0.0 a 0.0]^c-e^

^*^differences between baselines (p < 0.05).

^†^differences between baseline and 12 weeks for the control group (p < 0.05).

^‡^two different conditions from each other (p < 0.05).

^§^different conditions from each other between follow-up comparisons (p < 0.05).

^¶^differences between baseline and 12 weeks for the intervention group (p < 0.05).

^a^mean and standard deviation; maximum score of 13 (symptoms of DPN).

^b^median and interquartile interval; maximum score of 10 (tactile and vibration sensitivity, calcaneal reflex and foot inspection).

^c^mean and standard deviation; maximum of 100% (% of confidence referred by the patients).

^d^median and interquartile interval; muscle function test gradation (Kendall et al., 2005):

5:holds test position against maximal resistance.

4:holds test position against moderate resistance.

3:holds test position against gravity.

2:able to move through full ROM with gravity eliminated.

1:no visible movement, palpable or observed tendon prominence/flicker contraction.

0:no palpable or observable muscle contraction.

^e^Classification of foot function (Palmer and Epler, 1990):

4:normal functionality.

3:reasonable functionality.

2:little functionality.

1:absent functionality.

Stride velocity (not included in the tables) was not statistically different between groups and assessments (group effect: p = 0.57; time effect: p = 0.13; interaction effect: p = 0.61). Baseline velocity was 1.06 m/s (0.16 m/s) in the CG and 1.09 m/s (0.14 m/s) in the IG; after 12 weeks it was 1.04 m/s (0.12 m/s) in the CG and 1.05 m/s (0.14 m/s) in the IG. It neither differed in the follow-up assessment (p = 0.08), when it reached 1.11 m/s (0.15 m/s).

### Primary outcome and plantar pressure secondary outcomes

#### Primary outcome

After the IG treatment period, an increase was observed in peak pressure under the six foot areas and interaction effects were found at midfoot (Table 2). Interestingly, significant reductions appeared in the IG after the follow-up period, especially under the midfoot and the lateral forefoot.

#### Secondary outcomes

All in all, however, the intervention does not seem to have yielded significant changes in plantar pressure distribution after 12 weeks. ANOVA two-way analysis showed significant interaction effects for increase in outcomes of TPP delay at the heel and anticipation at the lateral forefoot (Table 2). Decrease in mean velocity of COP displacement presented an interaction effect under the midfoot and for total foot area (Table 2). Similarly to the primary outcomes, PTI displayed interaction effects at the midfoot (Table 2). Time effects were also observed in the IG. TPP was anticipated at the lateral forefoot, and delayed over the heel. After 12 weeks, there were increases in PTI in all foot regions, significant in the medial forefoot and hallux. The heel and toe areas displayed a medium effect size, and also showed increased PTI values after 12 weeks. An overall increasing tendency in PP was found in all foot regions after 12 weeks, but the increase was statistically significant only over the hallux.

As seen in Table 2, in the CG, PTI variations after 12 weeks of traditional medical care were not statistically significant, and a reverse tendency was observed at the midfoot and toes, as PTI decreased. A similar pattern was observed for PP, with a reverse tendency at the midfoot and toes. COP mean velocity also showed a non-significant reverse tendency over the heel, midfoot, and hallux.

Within the IG, comparisons between baseline, 12 weeks, and follow-up (ANOVA one-way) showed that all outcomes returned to baseline, except for TPP at the lateral forefoot (Table 2) and COP mean velocity (Table 2). As observed for PP, interestingly, significant PTI reductions under the midfoot and the lateral forefoot appeared in the IG after the follow-up period. It seems that COP changes were maintained after the follow-up period, and PP and PTI decreased again in the follow-up period after an initial increase (end of intervention), as if the foot-ankle complex had learnt how to manage the more articulated roll-over process.

### Secondary outcomes: kinetic and kinematic variables

There was an interaction effect for the total ankle range of motion and for the ankle angle at the end of the stance phase, where the CG showed decreasedresults after 12 weeks (Table 4). In the IG, there was a time effect of decreased peak ankle extensor moment after 12 weeks of intervention, which remained different from baseline until after follow-up.

**Table 4:** Mean, standard deviation and effects of intervention and follow-up on kinetics and kinematics of ankle

**Kinetic and kinematic**									
	**Control group**		**Intervention group**			**Intervention effect**		**Follow-up effect**	
	**Baseline (*a *) n = 29**	**12 weeks (*b *) n = 29**	**Baseline (*c *) n = 26**	**12 weeks (*d *) n = 26**	**24 weeks (*e *) n = 26**	**Effect size**^***-b-d ***^**(Cohen’s d)**	**p**^***a-b-c-d ***^**(interaction)**	**Efect size (Cohen’s d)**	**p**^***c-d-e***^
Sagittal ankle range of motion of stance phase (°)	22.5^†^ (3.5)	18.9^†^ (4.1)	20.8 (4.9)	20.8 (3.3)	19.6 (2.1)	0.53 (medium)	0.001	0.02 (small)^c-d^	0.14
								0.31 (small)^c-e^	
Sagittal ankle angle at the end of propulsion phase (°)	-8.4^†^ (5.8)	-5.5^†^ (3.9)	-7.5 (6.4)	-7.5 (4.6)	-7.8 (4.2)	0.46 (medium)	0.02	(small)^c-d^	0.91
								0.05 (small)^c-e^	
Sagittal peak of extensor moment of ankle ~20% stance phase (%BW.h)	-0.8 (0.2)	-0.7 (0.3)	-0.9^‡¶^ (0.4)	-0.7^‡^ (0.3)	-0.7^¶^ (0.3)	0.17 (small)	0.63	0.37 (small)^c-d^	0.02
								0.45 (medium)^c-e^	
Sagittal peak of flexor moment of ankle ~80% stance phase (%BW.h)	8.1 (0.7)	8.2 (0.4)	7.9 (0.9)	8.2 (0.9)	8.3 (0.4)	0.09 (small)	0.63	0.25 (small)^c-d^	0.17
								0.45 (medium)^c-e^	

^†^condition *a* is different from *b* (interaction and time effects, p < 0.001).

^‡^condition *c* is different from *d* (time effect, p < 0.01).

^¶^condition *c* is different from *e* in follow-up comparisons.

Letters a, b, c, d and e represent the groups conditions.

### Secondary outcomes: clinical variables

Compared to the baseline data, an expected significant increase in muscle function was observed in the flexor digitorum brevis, interosseous, tibialis anterior, and triceps surae in the IG after 12 weeks; the function of the same muscles worsened significantly in the CG, except for the triceps surae (Table 3). After the follow-up period, the IG returned to baseline as for the flexor digitorum brevis, lumbricals, interosseous, and tibialis anterior. The other muscles (extensor digitorum longus and brevis, extensor hallucis longus and brevis, flexor hallucis brevis, and triceps surae) also exhibited a worsened function, but the differences between 12 weeks and the follow-up period were not statistically significant.

All functional tests, except for ankle extension, showed a difference between groups after 12 weeks, which points to an improvement in the IG and a worsening in the CG (Table 3). In intragroup comparisons, the IG improved in all functional tests, while the CG showed a worsening for toes flexion. For toes extension it should be noted that both groups were different at baseline. At follow-up, an improvement was found for toes and ankle extension, but not for toes and ankle flexion.

As for the MNSI questionnaire, foot physical examination and ABC score (Table 3), there was no significant difference between groups in any of the assessments. In the IG , there was a reduction of 2 points in the MNSI questionnaire, with a medium effect size, which remained after the 12 week follow-up. This means that there was an improvement in the clinical condition of the patients. A reduction was also observed of the score for the physical examination of the feet at the follow-up assessment as compared to the corresponding scores after 12 weeks, but there was a significant improvement in the ABC score.

## Discussion

As a global interpretation, the results suggest that the proposed physiotherapy intervention in patients with DPN modestly changed the foot rollover to a more physiological process, supported by some improvement in dynamic pressure distribution, improvement of ankle extension moment and a better functional condition of the foot and ankle muscles.

### Primary outcomes

The expected reduction in forefoot PP was not observed at the end of the treatment, although our findings suggest a better foot rollover; rather, forefoot PP was significantly reduced after the follow up period, as was also noticed for midfoot PP. To better understand whether changes in the primary outcomes have to be interpreted as a sign of improvement or worsening of DNP overall foot function, intervention effects are hereby discussed in more detail, with reference to all the investigated variables grouped as biomechanical and clinical variables, and with respect to the main temporal phases of the rollover process.

### Effects of the intervention on biomechanical variables (plantar pressure, kinematic and kinetic variables)

In the heel strike phase, two important findings were observed. First, the intervention provided a delay in TPP at the heel (interaction effect), which means a lower rate of loading and better shock absorption in this gait phase. The second important change was the increase in PTI over the heel (medium effect). Since PP over the heel did not increase, the higher PTI was likely due to the delay in TPP occurrence and to a longer stance over this foot area. Since the protocol included exercises that involved proximal muscles as well (e.g. walking exercises), reduced impact at heel strike can be a consequence of the improved performance of hip flexors, hamstrings, and quadriceps [36], which were trained during the walking exercises. Softening of the heel impact is a positive outcome because a proper positioning of the heel at the initial ground/foot contact influences the positioning of the next foot segments during the midstance and propulsion phases [8,10] and ensures an adequate foot rollover process.

In the midstance phase, the IG showed a slower COP trajectory at the midfoot and unchanged PP and PTI after the intervention., while the CG showed a decrease in midfoot PTI. DPN patients are known to poorly control foot-flattening and midstance phases , mainly owing to the impaired eccentric function of the tibialis anterior, which is responsible for decelerating the forefoot until it touches the floor [17,18,45]. Besides, DPN patients have straight COP trajectory from the heel toward the medial forefoot, with shorter COP excursion in the medial-lateral direction [7]. Midfoot COP, PTI and PP results suggest a better control of the foot-flattening and midstance phases. The improvement in the foot-flattening phase after the intervention is also supported by the decreased ankle extensor moment, the improvement in the ankle dorsiflexion (functional tests) and in the function of the tibialis anterior (manual muscle function test).

At baseline, the TPP of the medial and lateral forefoot occurs simultaneously in both groups, which denotes an altered forefoot rollover, as the lateral region should make ground contact earlier than the medial area [10,11]. The anticipation of TPP at the lateral forefoot after 12 weeks of training (interaction effect) indicates a more physiological foot rollover, suggesting an improvement in foot mobility in the transverse plane. As demonstrated by Rao et al. [8], patients with DPN exhibit a loss of pronation and supination of the foot, which is imperative to allow for a proper loading transfer from the heel to the lateral forefoot. A limitation of the present study is that the mobility of the small foot joints could not be assessed. To confirm the hypothesized improvement in foot mobility in the transverse plane, further studies should address intrinsic foot mobility.

Considering again the primary outcome (PP) related to the forefoot areas, there was a group and time effect after 12 weeks, but no interaction effect, and both groups were different at baseline. Basically, the results are inconclusive as whether the proposed intervention did induce a relevant change in loading over forefoot PP. In any case, DPN prevention should not aim at merely reducing PP over the forefoot, mostly because this is not an optimal variable for describing time changes during the whole process of foot rollover. It only represents vertical loading during a very short time of the stance phase. Obviously, attention must be paid to keep plantar pressures under the proper risk threshold, although no cutoff value consensus for ulcer risk has been established yet [45].

The toes and hallux are known to have reduced participation in the walking of patients with DPN, reflected by a shorter COP excursion in the anteroposterior direction [7]. This is usually attributed to foot and ankle joint restrictions [3,8] and marked weakness of intrinsic foot muscles [19]. If the toes and hallux are no longer active due to DPN, the forefoot has to take over the function of propelling the body forward during walking and becomes overloaded [10], which has been associated with ulcer formation. The increased PP and PTI over the hallux, as well as the medium effect of the increase in PTI over the toes, were highly relevant to the foot function during the rollover process. The stretching exercises and walk training were efficient in improving hallux and toe mobility and function, reflected in the redistribution of plantar pressure. In addition, the increase in the score for the interosseous muscle function reasonably indicates a positive effect of the proposed strengthening exercises on toe function.

### Effects of the intervention on clinical variables

All in all, the foot and ankle complex as assessed by clinical tests (manual muscle function and repetition of movements) showed an improvement of the intrinsic and extrinsic muscle function, especially in ankle dorsiflexion (tibialis anterior), toes flexion (flexor digitorum brevis) and of interosseous muscles. The main differences between the groups were observed after 12 weeks. Ankle flexion and toes movements are expected to become impaired throughout the course of the disease. Training these functions preventatively may slow down the prognosis of the diabetes chronic complications. The gain in these functions can explain the changes in the pressure variables in foot rollover observed after 12 weeks of intervention, such as the decreased COP velocity at midfoot, the increased PTI in toes area, and the better control of forefoot contact.

The signs and symptoms of DPN had changed after the intervention (MNSI questionnaire score). The IG referred fewer symptoms after the intervention compared to the baseline condition. There was a slight worsening in the score of the physical examination of the feet in the control group after 12 weeks, and a small difference (1.5 points) between both groups after 12 weeks. This difference may indicate a worsening of tactile or vibration perceptions. Since twelve weeks is a relatively short period for structural deformities to set in, this change is likely to have occurred at the somatosensory level.

### Limitations and clinical implications

The main limitation of this clinical trial is the lack of follow-up for the CG. Therefore, follow-up results could not be compared between CG and IG. Besides, there was no control over patients’ compliance regarding home exercises during this period.

Previous studies do not describe the foot rollover changes after such a specific set of exercises for foot and ankle [24-27]. Usually, attention is mostly given to PP and PTI reduction as a target to reduce the risk of ulceration, but these variables should be analyzed together with several other risk factors [4,5,36]. This study also showed time-related changes during the complex task of foot rollover, among the most representative variables of foot loading transfer and management, and that can help better interpret the changes in PP and PTI variables.

The study population sample was composed mostly of non-severe neuropathic patients, i.e., subjects who have not lost all their sensitivity and muscle functions. The protocol described herein can be applied to every patient with or without diabetic neuropathy, regardless of the stage of the disease, the only exception being patients with tissue damage at the time of the exercise. However, the effect of this intervention has not yet been studied in severe neuropathic patients, with or without previous ulceration. Before implementing this type of intervention, a risk analysis is recommended to verify patient safety and compatibility of the exercise therapy with on-going treatment.

Lastly, the crucial role of preventive actions in neuropathic patients should be highlighted, as complications in muscles and joints occur over the long term, and it is of great importance to preserve and maintain their integrity. For incipient and moderately impaired patients with DPN, the proposed exercises are easy to perform at home, as opposed to general purpose exercises that need supervision (24,27). Stronger patient motivation and remote monitoring would certainly help patients reach their goals. Until now, most therapies have been applied only after ulceration and amputation, with relative success; only a few have been studied before such events.

## Conclusion

The combination of stretching, strengthening, and functional foot and ankle exercises provided modest changes in foot rollover. Pressure redistribution occurred in foot areas (heel, lateral forefoot, hallux, and toes) that are known to exhibit reduced participation in patients with DPN [7]. The lower velocity of COP displacement, without an increase in walking velocity or contact time, also indicates a dynamic loading pattern that shows better involvement of the whole foot**.** This change toward a more physiological pattern, together with foot and ankle function improvement, entails a better foot-to-floor interaction. Considering that DPN is a long-term disease, preventive actions such as this exercise protocol can be prescribed for this population as a complementary intervention. To maintain the obtained benefits longer, the periodic repetition of the intervention is recommended.

## Competing interests

The authors declare that they have no competing interests.

## Authors’ contributions

ICNS, CDS, RW, ACP and RHH were responsible for designing the study. CDS, CG, LPC and MKB were responsible for data analysis. All authors were responsible for discussing and interpreting the results and writing the final version to be published. ICNS, CDS, and ACP acted as trial coordinators. All the authors have approved the final manuscript. All authors read and approved the final manuscript.

## Pre-publication history

The pre-publication history for this paper can be accessed here:

http://www.biomedcentral.com/1471-2474/15/137/prepub

## Supplementary Material

Additional file 1: Table S1 Description, execution, and progression parameters of the exercises included in the intervention protocol. Click here for file

Additional file 2: Figure S2 Online only Figure 1 Example of exercises for **(a)** passive stretching of flexors and extensors of toes and hallux and **(b)** strengthening of ankle inversors, eversors and flexors. Online only Figure 2 – Examples of exercises for **(a)** balance training in unstable surface and **(b)** stretching of triceps surae. Online only Figure 3 – Example of exercises for **(a)** strengthening of tibialis anterior and triceps surae and **(b)** strengthening of intrinsic foot muscles using different materials. Click here for file
