# Supplementary material for: Effects of strengthening, stretching and functional training on foot function in patients with diabetic neuropathy: results of a randomized controlled trial
Source: BMC Musculoskelet Disord. 2014 Apr 27;15:137. doi: 10.1186/1471-2474-15-137 (PMC4031603; doi:10.1186/1471-2474-15-137)

Online only Figure 1 – Example of exercises for (a) passive stretching of flexors and extensors of toes and hallux and (b) strengthening of ankle inversors, eversors and flexors.

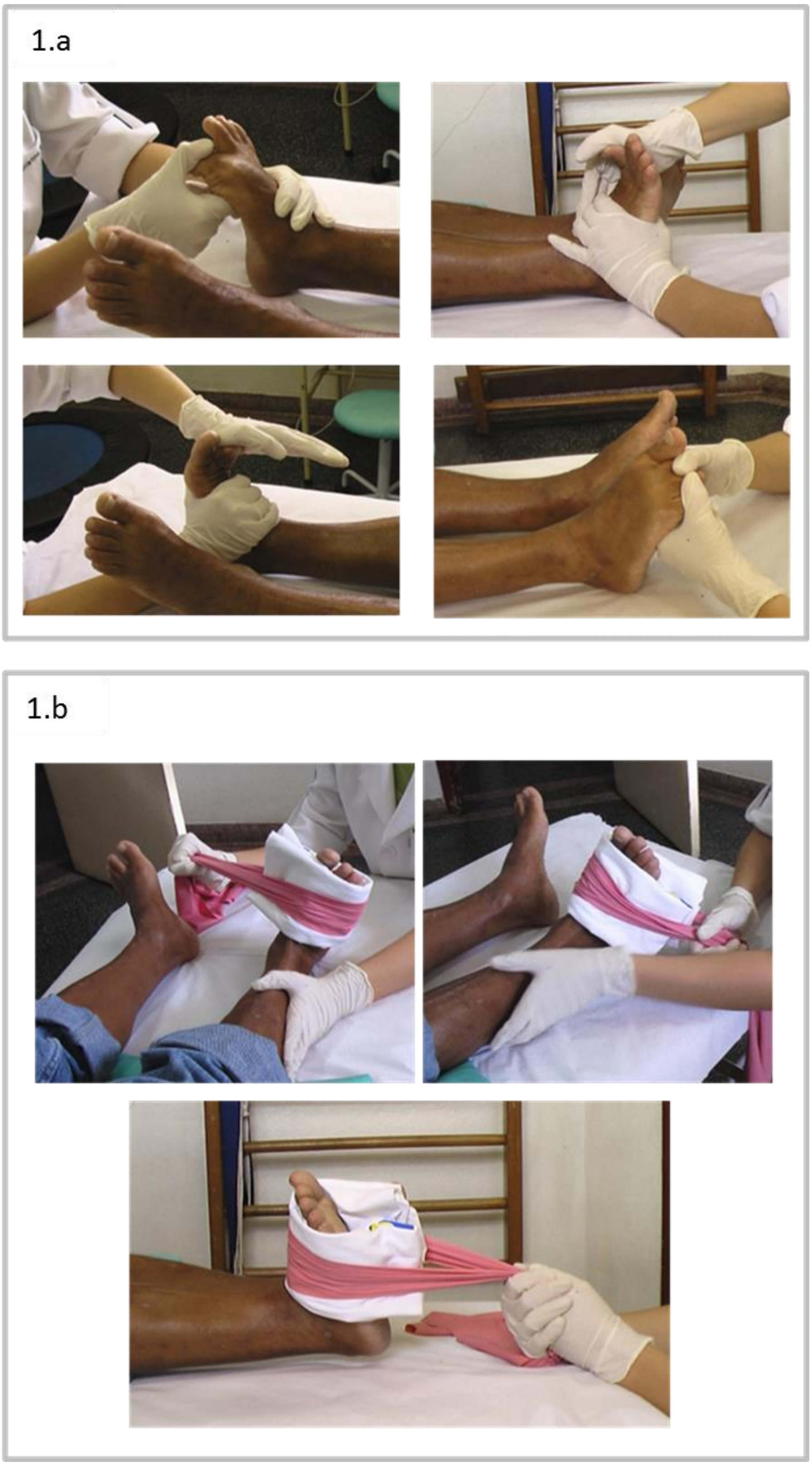

Online only Figure 22 – Examples of exercises for (a) balance training in unstable surface and (b) stretching of triceps surae.

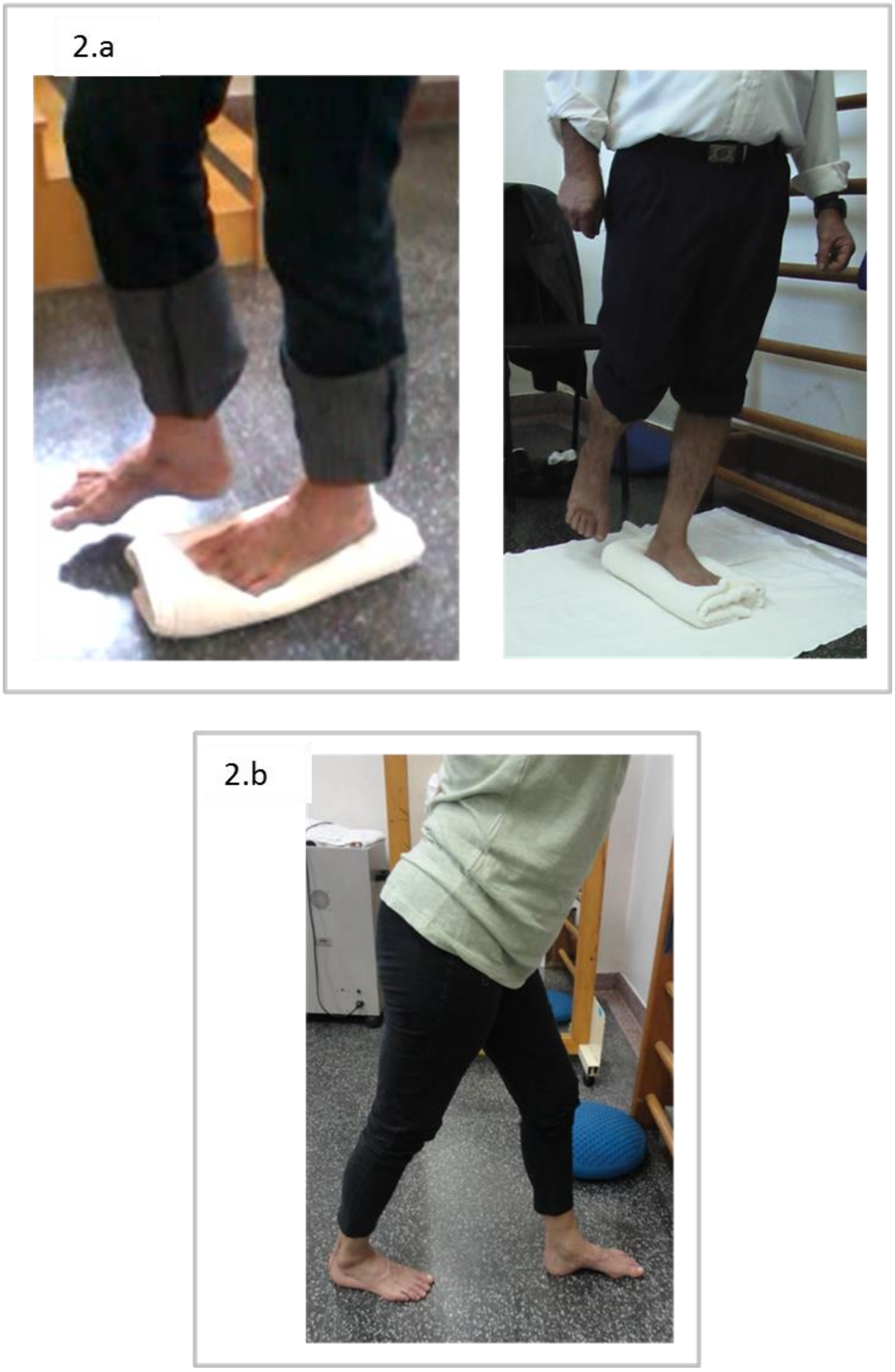

Online only Figure 3 – Example of exercises for (a) strengthening of tibialis anterior and triceps surae and (b) strengthening of intrinsic foot muscles using different materials.

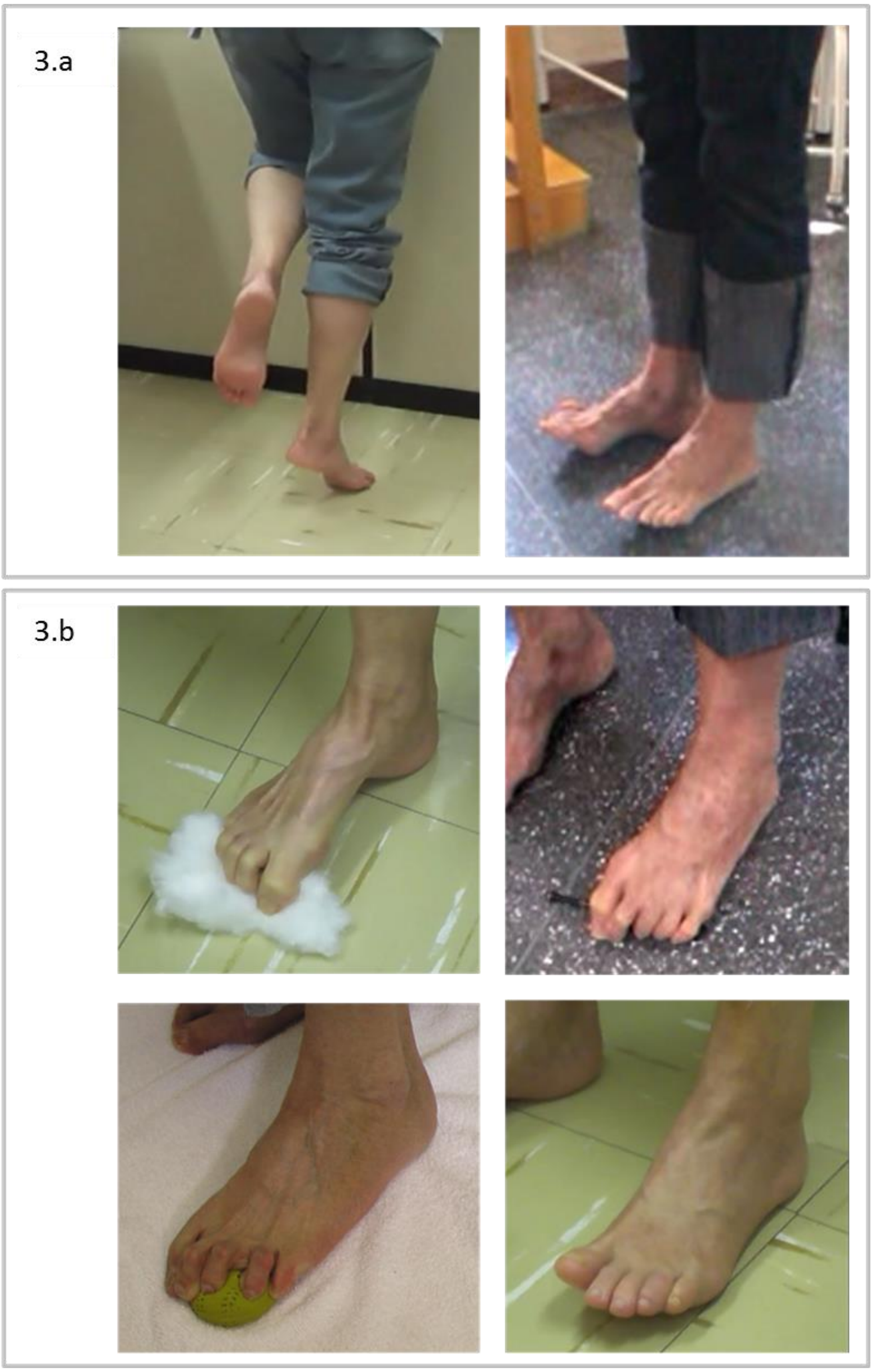

Supplement: Additional file 2: Figure S2 — Online only Figure 1 Example of exercises for (a) passive stretching of flexors and extensors of toes and hallux and (b) strengthening of ankle inversors, eversors and flexors. Online only Figure 2 – Examples of exercises for (a) balance training in unstable surface and (b) stretching of triceps surae. Online only Figure 3 – Example of exercises for (a) strengthening of tibialis anterior and triceps surae and (b) strengthening of intrinsic foot muscles using different materials. [file 1471-2474-15-137-S2.pdf]
